# Autopilot, Mind Wandering, and the Out of the Loop Performance Problem

**DOI:** 10.3389/fnins.2017.00541

**Published:** 2017-10-05

**Authors:** Jonas Gouraud, Arnaud Delorme, Bruno Berberian

**Affiliations:** ^1^Systems Control and Flight Dynamics Department, Office National d'Etudes et de Recherches Aérospatiales, Salon de Provence, France; ^2^Center of Research on Brain and Cognition (UMR 5549), Centre National de la Recherche Scientifique, Toulouse, France

**Keywords:** out of the loop, mind wandering, automation, performance, vigilance, psychophysiological measures, complacency

## Abstract

To satisfy the increasing demand for safer critical systems, engineers have integrated higher levels of automation, such as glass cockpits in aircraft, power plants, and driverless cars. These guiding principles relegate the operator to a monitoring role, increasing risks for humans to lack system understanding. The out of the loop performance problem arises when operators suffer from complacency and vigilance decrement; consequently, when automation does not behave as expected, understanding the system or taking back manual control may be difficult. Close to the out of the loop problem, mind wandering points to the propensity of the human mind to think about matters unrelated to the task at hand. This article reviews the literature related to both mind wandering and the out of the loop performance problem as it relates to task automation. We highlight studies showing how these phenomena interact with each other while impacting human performance within highly automated systems. We analyze how this proximity is supported by effects observed in automated environment, such as decoupling, sensory attention, and cognitive comprehension decrease. We also show that this link could be useful for detecting out of the loop situations through mind wandering markers. Finally, we examine the limitations of the current knowledge because many questions remain open to characterize interactions between out of the loop, mind wandering, and automation.

## Introduction

To continuously improve system safety, the critical systems industry makes extensive use of automation (Parasuraman, 1987; Billings, 1991; Sheridan, 1992; Degani and Heymann, 2000; Baxter et al., 2012). Automation has been introduced to answer performance and safety requirements in aircraft cockpits (Wise et al., 1994), in cars (Naujoks et al., 2016), and in power plant consoles (Cummings et al., 2010). Since the 1980s, designers integrated multiple modes of automation, allowing pilots to fly in autopilot mode. The automated mode is now able to maintain an altitude, fly to a point, or perform a landing, all without any human intervention (Wiener, 1988). Cars are currently going through the same revolution, as level 2 automation is being deployed—autopilots manage the car's trajectory while human supervision is still needed. At the same time, the industry is conducting studies of level 3 automation—no human intervention or supervision required (Ackerman, 2017). Unfortunately, if implementing higher levels of automation can improve the efficiency and capacity of a system, it also introduces difficulties for human operators.

It is now well-accepted that traditional automation has several negative consequences for performance and safety, a set of difficulties which are called out of the loop (OOTL) performance problem. The OOTL phenomenon corresponds to a deterioration of the operator's attention when interacting with highly automated system. The terms “total confusion” (Bureau d'Enquête et d'Analyse, 2002, p. 167; National Transport Safety Board, 1975, p. 17), “surprise effect” (Bureau d'Enquête et d'Analyse, 2012a, p. 10, Bureau d'Enquête et d'Analyse, 2016, p. 44) or “no awareness of the current mode of the system” (Bureau d'Enquête et d'Analyse, 2012b, p. 178) indicate a similar process—a mental state where the operator has lost his or her situation awareness and is not able to monitor the system efficiently. OOTL, which constitutes a human-machine miscommunication, has been pointed out as a cause of many accidents of various scales (Billings, 1991; Endsley and Kiris, 1995; Molloy and Parasuraman, 1996). Human-machine miscommunication describe situations where an operator or a machine “obtains an interpretation that she believes is complete and correct, but which is, however, not the one that the other speaker intended her to obtain” (McRoy, 2017). Miscomprehension can create accidents or drive operators to reject automation. For example, power plant operators declared to Andersson (2008) that they generally avoided higher automation level use because they “don't know what it is doing.” When the Federal Aviation Administration of the United States investigated the accident of the Eastern Airlines L-1011, which crashed during clear weather and with no apparent causes, the investigation concluded that the crew was focused on a red light in the cockpit and didn't notice that the autopilot had disengaged and that the plane started slowly going down (Federal Aviation Authority, 1972). At an operational level, the OOTL performance problem induces a performance decrease when trying to transfer manual control over the system (Berberian et al., 2012). Amongst other problems, an operator that is OOTL might take longer or be completely unable to detect an automation failure, decide if an intervention is needed, and find the adequate course of action. In the current context of the continued increase in automation, it is crucial to understand the sources of human–system interaction difficulties.

Although the OOTL performance problem represents a key challenge for system designers, it remains difficult to characterize and quantify after decades of research (Bainbridge, 1983; Baxter et al., 2012). Some researchers have pointed out vigilance failure as a key component of OOTL situations (Sarter and Woods, 1995b; Amalberti, 1999). Reports of incidents in aviation have notably illustrated the role of vigilance failure in human error. For example, Mosier et al. (1994) examined NASA's Aviation Safety Reporting System (ASRS) database and found that 77% of the incidents in which over-reliance on automation was suspected involved a probable vigilance failure. Similarly, Gerbert and Kemmler (1986) studied German aviators' anonymous responses to questionnaires about automation-related incidents and reported failures of vigilance as the largest contributor to human error. Nowadays, there is some consensus for the existence of a degradation of human operator vigilance in interaction with highly automated system (see, for example, O'Hanlon, 1981; Wiener, 1987; Strauch, 2002).

In this review, we aim to improve our comprehension of the OOTL performance problem and the related vigilance failure. In particular, we aim to explore the relation between the vigilance failures as observed in OOTL and the mind wandering (MW) phenomenon. MW is the human mind's propensity to generate thoughts unrelated to the task at hand (Christoff, 2012; Stawarczyk et al., 2012). MW is a fuzzy concept referring to the human mind's propensity to experience a variety of thoughts, which can be categorized along several dimensions. We will here use the term “mind wandering” to point out guided/unguided, internally/ externally generated and spontaneous/intended thoughts unrelated to the task at hand. Regardless the exact properties of these thoughts, the MW phenomenon diverts attention from immediate goals while the subject can be aware of it or not (Golchert et al., 2016; Seli et al., 2016). An individual who is MW is at least partly decoupled from his or her environment and show little to no reaction to external stimuli (Schooler et al., 2014). In brain imaging studies, MW is characterized by the activation of the Default Mode Network, a widely distributed brain region comprised of medial prefrontal cortex and the posterior cingulate cortex (Mason et al., 2007; Christoff et al., 2009; Christoff, 2012; Konishi et al., 2015). Even though MW is thought to facilitate prospection, introspection and problem solving (Smallwood and Schooler, 2006), performance drops in numerous tasks has been observed during MW episodes (He et al., 2011; Galera et al., 2012; Schad et al., 2012; Bastian and Sackur, 2013; Casner and Schooler, 2013, 2015; Yanko and Spalek, 2014; Berthié et al., 2015). Several aspects outline a possible role of MW in OOTL in highly reliable automated environments. This paper reviews the literature related to both MW and OOTL performance problem as it relates to automation. We investigate the possibility of a link between MW and OOTL by reviewing how features of both phenomena bridge the two together. Far from being only theoretical, we highlight how such a link could help both MW and OOTL research in practice. Finally, we analyze perspectives to go further toward understanding and detecting both phenomena.

## Mind wandering to complete OOTL theories

Multiple studies have showed that MW affects us all. The time we spend experiencing MW varies from 24 up to 60% depending on the study—40% in Schad et al. (2012); 47% in Killingsworth and Gilbert (2010); 24 and 31% in Bixler and D'Mello (2014); 30% in Kane et al. (2007); and 60% in Kam et al. (2011). This phenomenon has three major features: it is experienced by everybody (Killingsworth and Gilbert, 2010), it influences our behavior and attention toward external stimuli (He et al., 2011), and it can take place either intentionally or unintentionally (Smallwood and Schooler, 2006; Seli et al., 2016). All of those aspects pose a safety risk for any critical task requiring sustained attention, such as supervising automated systems.

MW is sensitive to multiple task characteristics. MW appears when the subject performs monotonous tasks (Eastwood et al., 2012). Familiar stimuli have been shown to increase MW (Bastian et al., 2017), while easier or longer tasks were also associated with more frequent MW episodes (Thomson et al., 2014; Smallwood and Schooler, 2015). MW might actually help to cope with boredom (Schooler et al., 2014). Boredom arises when people are unable to engage in satisfying activities while blaming their environment for it (Cummings et al., 2015). Several studies by Cheyne et al. (Cheyne et al., 2006; Carriere et al., 2008) point to the relationship between MW and boredom. Using questionnaires, they found a significant increase in everyday attentional failures for individuals more prone to boredom. Interestingly, Cummings et al. (2015) recently warned about a possible increase in boredom when integrating higher levels of automation. Moreover, MW related to automation was recently observed in automated systems. Casner and Schooler (2015) conducted a study where pilots were instructed to handle the approach—flight phase before landing—in a simulator by following beacons at altitudes given by the air traffic controller (ATC) officer. Probes inquired about their state of mind at predetermined times while the pilots had to report their position to the ATC officer. They observed that pilots were more prone to MW for higher levels of automation when they had no interaction with the system. Instead of planning the flight ahead, the pilots were inclined to think about unrelated matters. Although multiple studies have shown that monitoring is stressful and requires high levels of cognitive resources (Warm et al., 1996, 2008; Helton and Warm, 2008), vigilance theories do not explain such an increase in MW. On the contrary, could MW theories give a rational explanation in a monitoring environment?

### Complacency as a possible link between OOTL vigilance failure and MW

Automation technology has changed the very nature of operators' work. Pilots are now required to monitor systems for possible failures. Monitoring tasks request a constant attention from the subject in order to detect seldom and unpredictable events over prolonged periods of time. This fundamental function is called the sustained attention (Manly et al., 1999). Interestingly, several studies show that efficient sustained attention over hours cannot be achieved (e.g., Methot and Huitema, 1998). If research on vigilance suggests that time on task significantly decreases our ability to discriminate infrequent and unpredictable signals (Mackworth, 1948; Teichner, 1974; Parasuraman, 1979; Warm, 1984), then vigilance failures also encompass another reality when dealing with automation—that is, the complacency experienced by operators dealing with highly reliable automated systems (Parasuraman et al., 1993a; Cummings, 2004).

Overreliance or complacency is created by an uncritical reliance on the system leading to thinking of it as more competent than it actually is (Lee and See, 2004; Bahner et al., 2008). Operators working with systems that fail once every 10 million hours of use tend to underestimate the possibility of automation errors and overtrust the system (Amalberti, 2001; Parasuraman and Wickens, 2008). Because they have the feeling that the system does not require them to work efficiently, they instinctively lower cognitive resources allocated to monitoring (Thackray and Touchstone, 1989; Morrison et al., 1993). The first empirical evidence was the study by Parasuraman et al. (1993a). They tested non-pilot participants on a flight simulation task made of 2D compensatory tracking, fuel management, and system monitoring. In the multiple-task condition, the participants performed the tracking and fuel management tasks manually while the automation handled the system monitoring. In the single-task condition, the participants only had to supervise the automation in the system monitoring task. In both conditions, automation reliability was variable. The participants were responsible for detecting these failures, and they had to take over when there was a failure. Parasuraman et al. (1993a) observed that participants had a detection rate of over 70% when performing the engine status task manually (a baseline condition). Their detection rate substantially declined when performing the task in the multitask condition. Interestingly, the effect was absent when they were in the single task condition, suggesting that the allocation of cognitive resources plays a role in the complacency effect (Moray and Inagaki, 2000; Bailey and Scerbo, 2007). Congruently, operators make fewer eye movements to the raw information sources when using automation than under manual control (Metzger and Parasuraman, 2001; Bagheri and Jamieson, 2004; Wickens et al., 2005), reflecting an allocation of attention to other concurrent tasks. Furthermore, operators tend to less frequently visualize parameters in automation mode than under manual mode, thus blindly trusting the automation diagnosis (Lorenz et al., 2002; Manzey et al., 2006). In a low probability signal context, Manly et al. (1999) used a sustained attention to response task (SART, a GO/NOGO task), to demonstrate a striking positive correlation between signal probability and detection rate.

These results indicate that complacency could be closely linked to MW, as both complacency and MW divert cognitive resources away from the task at hand. Supervising ultra-reliable systems seems to encourage a decrease in cognitive resources allocated to the monitoring task. In this context, resources saved by automation, which should normally be used to plan the flight, would instead be directed toward task-unrelated thoughts. Therefore, complacency might lead operators to free cognitive resources and reallocate them to unrelated thoughts. This assertion is supported by an observed increase in MW in a low probability signal environment (Galera et al., 2012; Berthié et al., 2015; Casner and Schooler, 2015) and as one has been on task for a longer period of time (Teasdale et al., 1995; Smallwood et al., 2003, 2006; McVay and Kane, 2009; Thomson et al., 2014). Nevertheless, the exact direction of this link remains to be assessed. MW could also occur prior to complacency and modify its emergence, for example by lowering the level of confidence needed for the operator to become complacent. Further data is needed to take position.

### OOTL and MW: some similarities

#### Issues with decoupling of human observer from the task at hand

When designers integrate automation in systems, they often believe that it will only be a substitute to the human operator (i.e., substitution myth, see Woods and Tinapple, 1999). However, an important part of the literature has accumulated evidence against this view. Automation does not only simply perform tasks that were previously handled by humans. It also changes the complexity of the task and creates new issues, thus transforming the nature of human work. Operators give up their direct control over the system for a monitoring role in the supervisory control loop (Moray, 1986; Sheridan, 1992). These changes are far from trivial—direct control involves manual functions including process planning, decision making, selecting responses, and implementing strategies. At the same time, passive information monitoring only requires information sources to be scanned and compared to previously learned references. In an automated environment, operators can experience loss of manual skills (Baxter et al., 2012), a decreased sense of control (Berberian et al., 2012), and a feeling of distance from the system (Bainbridge, 1983). This distance disturbs the operator's involvement in the task. The same phenomenon of decoupling from the task is observed in MW. The operators' attention during MW is shifted from the immediate task toward unrelated concerns (Schooler et al., 2011). In other words, although the impact of MW and OOTL on operators' experience seems different, both influences start with a decoupling from the task. Moreover, both are equally threatening to safety in critical systems. For example, MW leads operators to forget to report as instructed (Casner and Schooler, 2015) and slows their adaptation to original tasks (Mooneyham and Schooler, 2013), whereas OOTL makes operators less responsive (Endsley and Kiris, 1995) and lowers their failure detection rate (Parasuraman and Riley, 1997).

#### Sensory attenuation problem

As defined by Endsley and Kiris (1995), OOTL is defined as the loss of one or more levels of situation awareness, which are perception (perceiving what is happening), comprehension (understanding the meaning of observed events), and projection (being able to think ahead). Given that perception guides both higher levels, its failure impacts the whole cognition. Several studies have shown a longer reaction time and lower detection rate following long automated periods. Endsley and Rodgers (1998) found that ATC officers showed poor performance in detecting conflicts within recorded traffic when they were passively monitoring the traffic. Willems and Truitt (1999) exposed that, in the same condition, ATC officers were slower to answer questions regarding traffic awareness and they recall less information as traffic load increases. In operational conditions, a lack of detection has led to tragic consequences. For example, the crash of the Mont-Saint-Odile (France) was due to a misunderstanding between the system and the pilots (Bureau d'Enquête et d'Analyse, 1992). During the landing procedure, the pilots selected the wrong units for the glide path, leading to a far steeper slope than expected. The cause was that the unit was not shown on the display but on the selection button. This accident demonstrates how operators can be impacted by OOTL and do not perform the usual checks on common procedures.

Similarly, MW involves a reduction in perceptual awareness of the task-relevant environment that lowers the subjects' ability to detect signals (Merat and Jamson, 2008; He et al., 2011; Blanchard et al., 2014), particularly when dealing with automation (Thackray and Touchstone, 1989). O'Connell et al. (2009) used a Sustained Attention to Response Task to demonstrate that alpha waves were higher during MW episodes in occipital scalp sites. Tasks analyzing selective attention, where one has to inhibit attention to parts of the environment in order to efficiently perform a task, suggest the involvement of alpha activity as a sensory suppression mechanism (Foxe et al., 1998; Foxe and Snyder, 2011), or similarly as reflecting pulsed-inhibition of ongoing cortical processing (Mathewson, 2011). Recently, both electroencephalography (EEG) and magnetic resonance imagery (MRI) have found alpha wave increases in supposedly deactivated regions by manipulating both the level of internally directed attention and the level of self-generated thought (Benedek et al., 2014, 2016), thus supporting the idea of alpha waves being a marker of inhibition. Taken together, these findings rule out the possibility that these effects could rely on sensory (bottom-up) processing of the cue and they suggest an endogenous inhibitory effect (top-down). During this time, the system and environment may change, hence increasing risks to the operator of having an out of date model of the situation. Without a proper perception of feedback and system modes, humans can lack the understanding that is mandatory to operate.

#### A human-machine interface communication problem

In addition to perception, cognitive comprehension may also be impacted by both phenomena. When automation fails or behaves abnormally, the operator is required to handle the difficulties alone. These cases have been well-documented in various domains, most notably flight deck and operating room automation (e.g., Sarter and Woods, 1995a,b; Degani and Heymann, 2000). Several fatal crashes and other incidents have been attributed to problems in the flight crew–automation interface (see for example Federal Aviation Authority, 1995). Sarter et al. (1997) referred to this as automation surprises, a point where the system behaves differently from what the operator expects. In laboratories, Wickens and Kessel (1977) demonstrated that operators removed from the system control show slower reactions and poor response accuracy. Carmody and Gluckman (1993) demonstrated that for complex task models, higher level of automation induced heavy losses of understanding. Taken together, these findings demonstrate that automation failures lead to a critical situation where the operator is OOTL and cannot initiate proper recovery actions.

Interestingly, similar understanding issues have been observed for MW. The subjects experience unconscious working memory transfer from the task at hand toward unrelated thoughts. Participants reading a text exhibited comprehension drops (Smallwood et al., 2008; Schad et al., 2012) and less reactions to text difficulties (Feng et al., 2013) during MW. Brain studies have shown activity uncorrelated to the environment during the same periods (Konishi et al., 2015). A decrement in external stimuli processing is particularly true within monotonous and uninteresting environments (Mosier et al., 1994). In the operational context, studies point to MW as a possible cause of many driving accidents (Galera et al., 2012), plane crashes (Casner and Schooler, 2013), and medical errors (van Charante et al., 1993), maybe due to a lack of a proper model of the situation in critical moments. Smallwood et al. (2007, 2011) developed the cascading model of inattention in order to offer an explanation. They suggest that the superficial deficit in information processing induced by MW would cascade and impair a deeper level of understanding and negatively impact the construction of an accurate situation model. The poor-quality model would then decrease the ability of the environment to hold the operator's attention, which in turn would decrease the quality of the model, and so on. Therefore, MW episodes would progressively impair the operator's situation model and their capability to handle seldom events. This degraded context could favor OOTL apparition and reveal MW important impact in critical situations.

### The exact nature of the link between MW and OOTL

After comparing MW and OOTL on multiple aspects, a question arises: how can they be linked? Casner and Schooler (2013) highlighted the blurry situation of pilots left with spare time and no guidance about how to actively monitor the automation. This spare time could encourage the operators to think about unrelated concerns and this would drive them away from important matters, such as their current position or the mode of the system. Without knowledge of the situation, OOTL risk rises, and threatens operations.

We suggest that MW and OOTL could interact through working memory. When experiencing MW, task-unrelated thoughts flood working memory (McVay and Kane, 2010). Depending on the individual's working memory capacity, MW thoughts might fully occupy working memory capacity, preventing new resources from being allocated to the ongoing task. As the observed vigilance decrement will lower available working memory, full capacity may be reached even more quickly within highly automated environments. At the same time, complacency could drive the operator to lower the amount of working memory capacity allocated to the task. The working memory capacity freed by complacency would be promptly used for more unrelated thoughts. Our framework is supported by various results examining the relations between MW—working memory and OOTL—working memory. Examining the trial-by-trial co-occurrence of MW and performance declines during a working memory span task, Schooler et al. (2014) found that MW precedes poor performance. Our framework states that when filled with task-unrelated thoughts, working memory capacity cannot cope with new cognitive needs. Then operators experience a drop in performance. Similarly, maintaining a good situation awareness—closely linked to whether one is OOTL (Kaber et al., 2000)—requires working memory capacity through the active manipulation and use of information (Durso et al., 1999). When executive resources are used by MW, the individual will see her situation awareness decrease, leading to a higher risk of being OOTL.

Nevertheless, the link between MW and OOTL remains unclear. Characterizing its features could help to both better define OOTL and understand some of the situations that have led to tragic accidents. To achieve this goal, MW markers could help study OOTL situations. We highlight some possible directions for research in the following sections.

## MW markers to study OOTL

### The need for online measures of OOTL

One of the biggest difficulties associated with automation is its insidious effect on situation awareness (SA) and performance. Several solutions have been designed to avoid OOTL. Among them, adaptive automation proposes to dynamically change the level of automation according to the value of a parameter. Workload and vigilance levels have already been used as automation triggers, with convincing results on SA and overall performances (Parasuraman et al., 1996; Mikulka et al., 2002). A possibility would be to directly use markers of SA to adapt the level of automation and avoid OOTL situations. Salmon et al. (2009) identified different categories of SA assessment methods, including freeze probe recall techniques, real-time probe techniques, post-trial subjective rating techniques, observer rating techniques, process indices, and performance measures. However, they are poorly suited for online use in operational environments. Most of them are disruptive and either necessitate task freezing (Endsley, 1988), post-trial assessment (Taylor, 1990), reports by an observer (Matthews and Beal, 2002), or direct questions to the subject (Durso et al., 1999). For example, one of the most used measures, the Situation Awareness Global Assessment Technique (Endsley, 1988), requires the pilot to halt the simulation and blank all displays. The pilot is then asked a series of questions to determine his knowledge of the current situation to determine his SA. The QUASA is another widely used measure of SA. The operator has to answer regular true/false probes followed by rating scales about his own confidence. Although this measure does not freeze the simulation, it diverts the operator's attention toward matters unrelated to the task. Critical systems cannot tolerate this impact on performances in real situations. Recent developments support psychophysiological markers to negate vigilance decrement, particularly within adaptive automation (Prinzel et al., 2003; Freeman et al., 2004). They create little intrusion for the subject, can record continuously (Eggemeier and Wilson, 1991; Kramer, 1991), and have already demonstrated a capacity to diagnostic on multiple levels; that is, arousal, attention and workload (Hancock and Williams, 1993; Harris et al., 1993; Parasuraman et al., 1993b; Boucsein et al., 2007). To help achieve better detection, recent findings in MW literature could help track OOTL. Many psychophysiological markers have already been extensively used in MW studies, covering a wide range of detection tools—from brain imaging to heart-rate and sudation, including oculometry. Therefore, it is necessary to examine the possibilities of tracking OOTL situations using MW markers.

### Self-report measures

MW markers are sorted using the triangulation classification among self-reports, physiological, and behavioral measures (Smallwood and Schooler, 2015). Self-reports regroup all of the subjective measures of MW. Most experiments use probes to determine periods when subjects are on-task or in MW (Smallwood et al., 2004; Gilbert et al., 2007; Braboszcz and Delorme, 2011; Uzzaman and Joordens, 2011; Feng et al., 2013). Although subjective reports have their limitation (Overgaard and Fazekas, 2016; Tsuchiya et al., 2016), they remain widely used to define an interval as MW or focused. Whereas, it may prove difficult for someone to report their level of vigilance, MW reports have demonstrated a high correlation with neurophysiological measures (Smallwood et al., 2008; Cowley, 2013). This robustness could prove to be useful when studying OOTL situations in laboratories but it would not be useful in operational environments. Nevertheless, other markers have demonstrated promising results and could be used with satisfying detection rates in the near future.

### Behavioral measures

Behavioral markers of MW come in a wide variety. Within this category, reaction time measurements take an important place. Multiple studies highlighted the progressively faster reaction time during MW, linking it to impulsive behavior (Smallwood et al., 2003, 2004, 2011; Cheyne et al., 2011). This parameter allows us to track the subject's attention without disturbing them. It contains much information, such as omissions—subject does not react to a stimulus although they were instructed to (see Bastian and Sackur, 2013)—and anticipations—reaction lower than 100 ms (see Hu et al., 2012). Cheyne et al. (2009) proved the robustness of the coefficient of variability—on a given interval, mean reaction time divided by its variability—to study MW in details (Bastian and Sackur, 2013; Esterman et al., 2013). Parallel to those results, subject accuracy is extensively used, whether it during trial to trial tasks (Braem et al., 2015; Durantin et al., 2015; Konishi et al., 2015) or during continuous monitoring, such as in a car simulator (He et al., 2011; Cowley, 2013; Yanko and Spalek, 2014). On the whole, behavioral markers can highlight performance decrements induced by MW in many different tasks. They can also be used for OOTL characterization; for example, reaction time to take manual control over a system (de Winter et al., 2014) or accuracy to detect automation failures (Metzger and Parasuraman, 2001). Unfortunately, these measures are also of limited use outside the laboratory. Reaction time is useful when the participants have to perform actions regularly whereas OOTL is mainly a problem when supervising highly automated systems where actions are seldom required. Given that accuracy measures the participants' shift from the goal, it is also limited to situations where the operator is already OOTL. Therefore, physiological measures could be useful to detect the dynamics of the problem.

### Oculometric measures

Oculometric measures allow us to derive different markers for potential use in detecting attentional lapses occurring during both MW and OOTL. Researchers demonstrated that, during visual tasks, pupil dilation occurs when subjects experience MW (Lowenstein and Loewenfeld, 1962; Yoss et al., 1970; Mittner et al., 2014). This behavior is correlated with norepinephrine activity in the locus coerulus (i.e., the LCNE system) and is thought to be linked with the role of surprise (Aston-Jones and Cohen, 2005; Gilzenrat et al., 2010; Jepma and Nieuwenhuis, 2011). MW is also accompanied by changes in gaze position (Grandchamp et al., 2014), a change in eye movement pattern (Smilek et al., 2010; He et al., 2011), blink count (Uzzaman and Joordens, 2011), and saccades (Bixler and D'Mello, 2014). Reading tasks highlighted differences in on and off-text fixations (Reichle et al., 2010; Bixler and D'Mello, 2015), reading speed (Franklin et al., 2011; Feng et al., 2013), especially related to text difficulty (Schad et al., 2012), within-word fixations, and reading regression (or going back a few words if one did not understand the sentence) (Uzzaman and Joordens, 2011). Given that vision is how we acquire most of our information, it is only logical that our eyes are highly influenced by lapses of attention. These advantages could contribute to make oculometry a necessity for OOTL detection.

### ECG and skin conductance measures

Heart rate and skin conductance have been used for a long time to detect periods of boredom (Smith, 1981) and they continue to be part of the latest developments. Their robustness allowed Pham and Wang (2015) to create a classifier which accurately identified lapses of attention during learning. They have also shown promising results when used to determine pilots' vigilance in real-time (Boucsein et al., 2007). The effects of boredom over amplitude and variability were assessed on both markers. Interestingly, Smallwood et al. (2004) reported similar effects when studying MW. Since MW may favor OOTL situations, heart rate, and skin conductance could also be used to study OOTL. Regrettably, it is possible that MW influence over the signal would be lost within operational environment because stress, movement, and temperature can also play a role in heart rate and skin conductance variations. Consequently, more studies will be required in this field.

### Neural markers

Neural markers of attention lapses are used to both detect MW and reveal its dynamics. Researchers have mostly used EEG or functional MRI (fMRI) to study those markers, with the notable exception of the HbO2 concentration using functional near-infrared spectroscopy (fNIRS) (see Durantin et al., 2015). EEG activity has a high temporal resolution and a relatively low cost (Luck, 2014), allowing its extensive use for MW research. MW influence on brain waves was suggested by EEG data with an accent on the alpha band (8–14 Hz), although the direction of the influence is still debated (O'Connell et al., 2009; Braboszcz and Delorme, 2011), and event related potentials (ERPs). Sensory attenuation has been observed on the visual component P1 and the auditory component N1 (Kam et al., 2011), while the lack of stimulus processing was shown using P3 (Schooler et al., 2011), N400 (O'Connell et al., 2009), and fERN (Kam et al., 2012). By contrast, fMRI has a fine spatial resolution but a poor temporal resolution and may be used to detect neuronal networks involved in MW in order to build a map of the wandering mind. Several studies have highlighted brain regions differently involved in the phenomenon, such as the default mode network (Mason et al., 2007; van den Heuvel and Hulshoff Pol, 2010), the executive network (Christoff et al., 2004, 2009) and the task-positive network (Mittner et al., 2014). Compared to other markers, neural markers of MW could not only answer the question of “when” OOTL occurs, but also the “why” and “how”. This could provide the OOTL performance problem with the physiological definition that it lacks.

MW research has identified an important set of markers to detect its occurrence. Due to the proximity with psychophysiological measures recently used in automation studies, these markers may also prove to be useful for OOTL research. However, many unknowns still remain regarding some aspects of both phenomena and the feasibility of their study within operational environments is uncertain.

## Limits to current approaches

The use of MW findings could be a huge step toward understanding and countering OOTL's deleterious effects on human performance. MW physiological aspects are for now far better apprehended than for OOTL, while its influence over performance is more precisely assessed, even though many parts of MW remain largely unknown and could limit the transposition.

### Different levels of MW

Generally, studies postulate that MW is a binary state, for example when questionnaires ask if the subject is in MW or focused (Braboszcz and Delorme, 2011; Smallwood et al., 2011; Bastian and Sackur, 2013; van Vugt et al., 2015). By contrast, the inattention hypothesis suggested by Smallwood (2011) proposes a gradual view of MW. They manipulated a corpus of text by inserting different types of errors, from pseudo-words (lower level errors) to inconsistent statements (higher level errors). During the experiment, those participants who experienced MW exhibited progressive gaze pattern modification depending on error level, supporting a graded nature of the phenomenon. This is in line with findings concerning response time, which mention a progressive acceleration of response times before MW reports (Smallwood et al., 2008; Smallwood, 2010). Cheyne et al. (2009) proposed a three-level model of MW by postulating that response time degradation—slowing, anticipation and omissions—could each correspond to a different level. This hypothesis is empirically confirmed by our ability to perform everyday tasks accurately in spite of MW. For example, driving is still possible with MW (Lerner et al., 2015; Qu et al., 2015) even though it does affect performance. This could also explain why operators can experience MW without systematic OOTL problems. Investigating this possibility will require changing paradigms. Whereas, the probes so far have asked the subject to report their state of mind in a binary fashion, we need to use a scale and compare its results to the evolution of psychophysiological markers. Eventually, taking this parameter into account could allow us to develop systems that are able to discriminate between levels of MW.

### Mind wandering and cognitive fatigue

It is now clear that MW during driving and piloting tasks decreases short-term performance, especially when the operator is moved to a supervising role. However, the long-term consequences of MW have not been assessed. We experience this on a daily basis—if it was detrimental to survival, there is little doubt that evolution would have removed it (Schooler et al., 2014). Therefore, what are the advantages of such a state of mind? Several papers have highlighted the benefits of MW for curiosity, social skills (McMillan et al., 2013), and creative problem solving (Zedelius and Schooler, 2015, 2016). Another possible advantage of MW could be linked to cognitive fatigue. Humans experience high levels of cognitive fatigue and stress when facing monitoring tasks in monotonous and repetitive environments (Thackray and Touchstone, 1989; Sarter et al., 1997; Warm et al., 2008). At the same time, it has been established that MW propensity increases as the task lasts (Esterman et al., 2013; Pham and Wang, 2015). Therefore, MW may be a mechanism that has been built to decrease cognitive fatigue. Boredom studies mentioned daydreaming as a strategy to cope with boredom within monotonous environments, such as driving, monitoring, or piloting (Davies, 1926; Harris, 2000). The best paradigm to investigate this theory would be to perform real-time tracking and suppress MW as soon as it is detected. Observing the results on mood, fatigue, and arousal could provide precious information about MW's advantages. Unfortunately this protocol is not possible for now due to MW low detection rates. However, the outcomes would be systems that are able to discriminate between intrusive MW episodes and useful ones, depending on the situation, such as flight phase or traffic density. These systems would reduce OOTL risks while benefitting from MW.

### Real-time detection of MW

When talking about MW research, a straightforward question is to ask if researchers can assess one's state of mind at a given moment. Such as, whether or not he or she is in MW? This possibility would offer countless possibilities to the study MW. It could, for example, highlight its triggers, assess its benefits, study its dynamic, and define the precise influence of environmental conditions. Recently, studies trying to perform such detection have flourished. They tend to use classifiers, programs that gather information to compare them to a reference and assess if the subject is MW or focused (Delorme et al., 2010). Detection rates are reported through kappa, which is a metric comparing an observed accuracy with an expected one, and included between 0 (random chance) and 1 (exact prediction). Given that reading is an activity where participants do not move much but interact extensively with their environment, it has been the first context used to perform MW detection. Using previous findings (Smallwood et al., 2004) on the influence of MW over galvanic skin conductance, Blanchard et al. (2014) reached a kappa of 0.22. The same kappa was obtained by Pham and Wang (2015) with heart rate variability. Finally, Bixler and D'Mello (2014, 2015) used oculometry during reading to build a classifier which reached 0.31. However, reading is not the only paradigm used for MW detection. Melinscak et al. (2014) asked the participant to pay attention or ignore some kinesthetic sensation. They developed a classifier using a passive brain-computer interface (BCI) with a kappa of 0.33, which is the best result so far among MW classifiers. Although using neuroimaging to monitor the participants' attention seems promising, artifacts on the EEG signal make online processing difficult.

### Multimodal classifiers

It is worth noting that, to our knowledge, all studies trying to perform MW or OOTL online detection did so with only one kind of measure—whether it was heart rate, oculometry, or EEG signal—with the notable exception of Boucsein et al. (2007). It may prove useful to research multimodal classifiers to see if the success rate can be increased. Nevertheless, combining measures would not necessarily result in better detection. Indeed, the main difficulty is to not only design accurate classifiers in order to obtain good prediction but also ensure that the classifiers are sturdy enough for it to be generalized across subjects and conditions. More particularly, high intra- and inter-subject variability make it difficult to build a robust classifier. Intra-subject variability describes the differences observed on one subject depending on their environment. Time, fatigue, and interest are parameters that could influence MW episodes frequency, length and deepness (Smith, 1981; Smallwood et al., 2004; Cummings et al., 2015). Grandy et al. (2013) demonstrated that each human has a stable alpha wave frequency that is independent from cognitive interventions. On the other hand, they observed important differences between subjects in this frequency band. Inter-subject variability often prevents us from building a robust model that is able to be generalized across subjects. One solution is to have the model adapt itself to the user, and then use markers and thresholds that are specific to each individual. However, this model would have a high cost, shortening its range of applications.

### Use MW detection within operational environment

Although experiments performed in laboratory conditions (e.g., reading and simulators experiments) have produced useful results, they were all performed in a controlled environment. Bixler and D'Mello (2014) have shown the possibility of performing experiments on actual users instead of experimental subjects, although only in a reading tasks. Within an operational environment, systems need to minimize any disruption from the detectors, especially in safety critical environments. Mkrtchyan et al. (2012) described an ATC interface designed to detect and counter lapses of attention using EEG, thanks to the officer sitting and the stable environment. However, it can be extremely difficult to achieve for pilots and drivers. Not only does the subject variably increase the difficulty to build robust classifiers but conditions of measures can also introduce much noise.

Some systems have recently been designed to overcome these issues. Addressing ease of implementation, dry electrodes measure EEG signal without need for skin preparation (Taheri et al., 1994). Although the signal-to-noise signal is lower and requires further improvement, it could be implemented in operational environments with little disruption for the user, especially if they already wear a helmet, such as jet pilots. Mullen et al. (2015) used this technology to design a wearable EEG system for online neuroimaging with promising results. Recent advances in high-tech industry could produce interesting results in a near future, such as MindRDR (This Place, 2016) or OpenBCI (OpenBCI, 2016). Proving that EEG is not the only promising brain signal measure, Khan and Hong (2015) used fNIRS recorded with a BCI to detect drowsiness with a success rate of ~84%. Oculometry has also been substantially improved over the past decade, producing efficient, small, and cheap devices. Systems have been proposed with several designs—head-mounted or deported—and they can be integrated in almost any preexisting system with efficient results. Scanella et al. (2015) showed that flight phases could be differentiated using an eye tracker while demonstrating a remarkable independence regarding inter- and intra-subject variability. Closer to vigilance research, Dehais et al. (2008, 2010) found that an embedded eye tracker allowed detection of gaze features during flight in both nominal and degraded conditions. Several studies have demonstrated the possibility of using EEG for vigilance monitoring in operational environments (Dussault et al., 2005; Jeroski et al., 2014). Cabon et al. (1993) gathered data from ECG put on long range aircrews and train drivers with the device attached on the seatbelts. Boucsein et al. (2007) recorded the same information—with a more invasive system—to design a flight simulator interface using adaptive automation. Their system could accurately react to varying levels of vigilance. However, the acceptability—which is defined as the capacity of the system to fulfill user's needs and be accepted for a regular use—was not evaluated during the experiment. Still, these results demonstrate the possibility of building better human-machine interfaces, which could potentially prevent many vigilance related accidents.

## Conclusion

The OOTL phenomenon has been involved in many accidents in safety-critical industries, as demonstrated by papers and reports that we have reviewed. In the near future, the massive use of automation in everyday systems will reinforce this problem. MW may be closely related to OOTL—both involve removal from the task at hand, perception drop, and understanding problems. More importantly, their relation to vigilance decrement and working memory could be the heart of their interactions. Still, the exact causal link remains to be demonstrated. Far from being anecdotal, such a link would allow OOTL research to use theoretical and experimental understanding accumulated on MW. The large range of MW markers could be used to detect OOTL situations and help us to understand the underlying dynamics. On the other hand, designing systems capable of detecting and countering MW might highlight the reason why we all mind wander. Eventually, the expected outcome is a model of OOTL–MW interactions which could be integrated into autonomous systems.

This system description echoes recent advances toward adaptive and communicative automation (Cassell and Vilhjálmsson, 1999; Sarter, 2000; May and Baldwin, 2009). Adaptive systems could detect and react to operators' state of mind, including mood, motivation, fatigue, or arousal. The signals sent, information displayed, and levels of automation could be adjusted by the system to maximize situation awareness and vigilance. These systems could detect MW and decide whether it should be stopped or allowed depending on the situation and the characteristics of the episode. Thus, the operator could benefit from MW's advantages while having a reduced risk of going on to OOTL. The benefits of keeping an operator always in the loop could demonstrate that humans can still be useful in safety favoring industries.

## Author contributions

All authors listed have made substantial, direct and intellectual contribution to the work, and approved it for publication.

### Conflict of interest statement

The authors declare that the research was conducted in the absence of any commercial or financial relationships that could be construed as a potential conflict of interest.

## References

[B1] Ackerman E. (2017). Cadillac Adds Level 2 Highway Autonomy With Super Cruise, in IEEE Spectrum Technology Engineering and Science News. Available online at: http://spectrum.ieee.org/cars-that-think/transportation/self-driving/cadillac-adds-level-2-highway-autonomy-with-super-cruise (Accessed July 19, 2017).

[B2] Amalberti R. (1999). Automation in aviation: a human factors perspective, in Aviation Human Factors, eds Garlan D. Wise J. Hopkin D. (Hillsdale, MI: Lawrence Erlbaum Associates), 173–192.

[B3] Amalberti R. (2001). The paradoxes of almost totally safe transportation systems. Saf. Sci. 37, 109–126. 10.1016/S0925-7535(00)00045-X

[B4] Andersson J. (2008). Levels of Automation and User Control—Evaluation of a Turbine Automation Interface. Chalmers: Nordic Nuclear Safety Research.

[B5] Aston-Jones G. Cohen J. D. (2005). An integrative theory of locus coeruleus-norepinephrine function: adaptive gain and optimal performance. Annu. Rev. Neurosci. 28, 403–450. 10.1146/annurev.neuro.28.061604.135709 16022602

[B6] Bagheri N. Jamieson G. A. (2004). Considering subjective trust and monitoring behavior in assessing automation-induced complacency, in Human-Automation Manufacturing Industry System: Current Trends and Practice, eds Vicenzi A. Mouloua M. Hancock O. A. (Mahwah, NJ: Lawrence Erlbaum), 54–59.

[B7] Bahner J. E. Hüper A.-D. Manzey D. (2008). Misuse of automated decision aids: complacency, automation bias and the impact of training experience. Int. J. Hum. Comput. Stud. 66, 688–699. 10.1016/j.ijhcs.2008.06.001

[B8] Bailey N. R. Scerbo M. W. (2007). Automation-induced complacency for monitoring highly reliable systems: the role of task complexity, system experience, and operator trust. Theor. Issues Ergon. Sci. 8, 321–348. 10.1080/14639220500535301

[B9] Bainbridge L. (1983). Ironies of automation. Automatica 19, 775–779. 10.1016/0005-1098(83)90046-8

[B10] Bastian M. Sackur J. (2013). Mind wandering at the fingertips: automatic parsing of subjective states based on response time variability. Front. Psychol. 4:573. 10.3389/fpsyg.2013.00573 24046753 PMC3763218

[B11] Bastian M. Lerique S. Adam V. Franklin M. S. Schooler J. W. Sackur J. (2017). Language facilitates introspection: verbal mind-wandering has privileged access to consciousness. Conscious. Cogn. 49, 86–97. 10.1016/j.concog.2017.01.002 28161598

[B12] Baxter G. Rooksby J. Wang Y. Khajeh-Hosseini A. (2012). The ironies of automation … still going strong at 30?, in Proceedings of ECCE 2012 Conference (Edinburgh).

[B13] Benedek M. Jauk E. Beaty R. E. Fink A. Koschutnig K. Neubauer A. C. (2016). Brain mechanisms associated with internally directed attention and self-generated thought. Sci. Rep. 6:22959. 10.1038/srep22959 26960259 PMC4785374

[B14] Benedek M. Schickel R. J. Jauk E. Fink A. Neubauer A. C. (2014). Alpha power increases in right parietal cortex reflects focused internal attention. Neuropsychologia 56, 393–400. 10.1016/j.neuropsychologia.2014.02.010 24561034 PMC3989020

[B15] Berberian B. Sarrazin J.-C. Le Blaye P. Haggard P. (2012). Automation technology and sense of control: a window on human agency. PLoS ONE 7:e34075. 10.1371/journal.pone.0034075 22479528 PMC3316600

[B16] Berthié G. Lemercier C. Paubel P.-V. Cour M. Fort A. Galera C.. (2015). The restless mind while driving: drivers' thoughts behind the wheel. Accid. Anal. Prev. 76, 159–165. 10.1016/j.aap.2015.01.005 25697452

[B17] Billings C. E. (1991). Human-Centered Aircraft Automation: A Concept and Guidelines. Available online at: http://ntrs.nasa.gov/search.jsp?R=19910022821 (Accessed July 23, 2014).

[B18] Bixler R. D'Mello S. (2014). Toward fully automated person-independent detection of mind wandering, in User Modeling, Adaptation, and Personalization (Springer), 37–48. Available online at: http://link.springer.com/chapter/10.1007/978-3-319-08786-3_4 (Accessed October 20, 2015).

[B19] Bixler R. D'Mello S. (2015). Automatic gaze-based user-independent detection of mind wandering during computerized reading, in Proceedings of the 23rd User Modeling and User-Adapted Interactions Conference (Dublin), 31–43. 10.1007/s11257-015-9167-1

[B20] Blanchard N. Bixler R. Joyce T. D'Mello S. (2014). Automated physiological-based detection of mind wandering during learning, in Intelligent Tutoring Systems (Springer), 55–60. Available online at: http://link.springer.com/chapter/10.1007/978-3-319-07221-0_7 (Accessed February 25, 2016).

[B21] Boucsein W. Haarmann A. Schaefer F. (2007). Combining skin conductance and heart rate variability for adaptive automation during simulated IFR flight, in Engineering Psychology and Cognitive Ergonomics (Springer), 639–647. Available online at: http://link.springer.com/chapter/10.1007/978-3-540-73331-7_70 (Accessed May 9, 2016).

[B22] Braboszcz C. Delorme A. (2011). Lost in thoughts: neural markers of low alertness during mind wandering. Neuroimage 54, 3040–3047. 10.1016/j.neuroimage.2010.10.008 20946963

[B23] Braem S. Coenen E. Bombeke K. van Bochove M. E. Notebaert W. (2015). Open your eyes for prediction errors. Cogn. Affect. Behav. Neurosci. 15, 374–380. 10.3758/s13415-014-0333-4 25588818

[B24] Bureau d'Enquête et d'Analyse (1992). Rapport Final Sur l'Accident Survenu le 20 Janvier 1992 à l'Airbus A320 Exploité par Air Inter Immatriculé F-GGED sur L'approche de l'aéroport Strasbourg-Entzheim. Bureau d'Enquête et d'Analyse.

[B25] Bureau d'Enquête et d'Analyse (2002). Rapport Final de l'Accident Survenu le 2 Octobre 1996 au Boeing 757 Exploité par Aeroperu vol Lima - Santiago. Bureau d'Enquête et d'Analyse, Paris.

[B26] Bureau d'Enquête et d'Analyse (2012a). Rapport Final de l'Accident Survenu le 1er Juin 2009 à l'Airbus A330-203 Immatriculé F-GZCP Exploité Par Air France vol AF 447 Rio de Janeiro - Paris. Bureau d'Enquête et d'Analyse, Paris.

[B27] Bureau d'Enquête et d'Analyse (2012b). Rapport Final sur L'incident Grave Survenu le 27 Février 2012 à l'Airbus A330 Exploité Par Air France Immatriculé F-GZCG sur le vol Antananarivo - Paris. Bureau d'Enquête et d'Analyse, Paris.

[B28] Bureau d'Enquête et d'Analyse (2016). Rapport Final Sur l'incident Grave Survenu le 24 Mai 2011 Sur le Dassault Falcon 7X, Immatriculé HB-JFN à Subang. Bureau d'Enquête et d'Analyse, Paris.

[B29] Cabon P. Coblentz A. Mollard R. Fouillot J. P. (1993). Human vigilance in railway and long-haul flight operation. Ergonomics 36, 1019–1033. 10.1080/00140139308967974 8404831

[B30] Carmody M. A. Gluckman J. P. (1993). Task specific effects of automation and automation failure on performance, workload, and situational awareness, in Seventh International Symposium on Aviation Psychology (Columbus, OH).

[B31] Carriere J. S. A. Cheyne J. A. Smilek D. (2008). Everyday attention lapses and memory failures: the affective consequences of mindlessness. Conscious. Cogn. 17, 835–847. 10.1016/j.concog.2007.04.008 17574866

[B32] Casner S. M. Schooler J. W. (2013). Thoughts in flight automation use and Pilots' task-related and task-unrelated thought. Hum. Factors J. Hum. Factors Ergon. Soc. 56, 433–442. 10.1177/0018720813501550 24930166

[B33] Casner S. M. Schooler J. W. (2015). Vigilance impossible: diligence, distraction, and daydreaming all lead to failures in a practical monitoring task. Conscious. Cogn. 35, 33–41. 10.1016/j.concog.2015.04.019 25966369

[B34] Cassell J. Vilhjálmsson H. (1999). Fully embodied conversational avatars: making communicative behaviors autonomous. Auton. Agents Multi Agent Syst. 2, 45–64. 10.1023/A:1010027123541

[B35] Cheyne A. J. Solman G. J. F. Carriere J. S. A. Smilek D. (2009). Anatomy of an error: a bidirectional state model of task engagement/disengagement and attention-related errors. Cognition 111, 98–113. 10.1016/j.cognition.2008.12.009 19215913

[B36] Cheyne J. A. Carriere J. S. A. Smilek D. (2006). Absent-mindedness: lapses of conscious awareness and everyday cognitive failures. Conscious. Cogn. 15, 578–592. 10.1016/j.concog.2005.11.009 16427318

[B37] Cheyne J. A. Carriere J. S. A. Solman G. J. F. Smilek D. (2011). Challenge and error: critical events and attention-related errors. Cognition 121, 437–446. 10.1016/j.cognition.2011.07.010 21861999

[B38] Christoff K. (2012). Undirected thought: neural determinants and correlates. Brain Res. 1428, 51–59. 10.1016/j.brainres.2011.09.060 22071565

[B39] Christoff K. Gordon A. M. Smallwood J. Smith R. Schooler J. W. (2009). Experience sampling during fMRI reveals default network and executive system contributions to mind wandering. Proc. Natl. Acad. Sci. U.S.A. 106, 8719–8724. 10.1073/pnas.0900234106 19433790 PMC2689035

[B40] Christoff K. Ream J. M. Gabrieli J. D. E. (2004). Neural basis of spontaneous thought processes. Cortex 40, 623–630. 10.1016/S0010-9452(08)70158-8 15505972

[B41] Cowley J. A. (2013). Off task thinking types and performance decrements during simulated automobile driving. Proc. Hum. Factors Ergon. Soc. Annu. Meet. 57, 1214–1218. 10.1177/1541931213571270

[B42] Cummings M. L. (2004). Automation bias in intelligent time critical decision support systems. Am. Institure Aeronaut. Astronaut. 2, 557–562. 10.2514/6.2004-6313

[B43] Cummings M. L. Gao F. Thornburg K. M. (2015). Boredom in the Workplace: a new look at an old problem. Hum. Factors 58, 279–300. 10.1177/0018720815609503 26490443

[B44] Cummings M. L. Sasangohar F. Thornburg K. M. Xing J. D'Agostino A. (2010). Human-System Interface Complexity and Opacity Part I: Literature Review. Cambridge, MA: Massachusetts Institute of Technology Available online at: https://www.researchgate.net/profile/Farzan_Sasangohar/publication/263315585_Humansystem_interface_complexity_and_opacity/links/0deec53accc4413fb2000000.pdf (Accessed October 18, 2016).

[B45] Davies A. H. (1926). The physical and mental effects of monotony in modern industry. Br. Med. J. 2, 472–479.

[B46] Degani A. Heymann M. (2000). Pilot-autopilot interaction: a formal perspective. Abbott 1, 157–168. 10.1207/s15327108ijap0902_3

[B47] Dehais F. Causse M. Pastor J. (2008). Embedded eye tracker in a real aircraft: new perspectives on pilot/aircraft interaction monitoring, in Proceedings from The 3rd International Conference on Research in Air Transportation (Fairfax, VA: Federal Aviation Administration). Available online at: http://mickael.causse.free.fr/medias/publis/Embedded%20eye%20tracker%20in%20a%20real%20aircraft%20new%20perspectives%20on%20pilot%20aircraft%20interaction%20monitoring.pdf (Accessed May 9, 2016).

[B48] Dehais F. Causse M. Pastor J. (2010). Toward the definition of a pilot's physiological state vector through oculometry: a preliminary study in real flight conditions, in Proceedings of HCI Aero. Available online at: http://mickael.causse.free.fr/medias/publis/HCI_dehais_causse_pastor.pdf (Accessed August 24, 2016).

[B49] Delorme A. Kothe C. Vankov A. Bigdely-Shamlo N. Oostenveld R. Zander T. O. (2010). MATLAB-based tools for BCI research, in Brain-Computer Interfaces (Springer), 241–259. Available online at: http://link.springer.com/chapter/10.1007/978-1-84996-272-8_14 (Accessed October 5, 2015).

[B50] de Winter J. C. F. Happee R. Martens M. H. Stanton N. A. (2014). Effects of adaptive cruise control and highly automated driving on workload and situation awareness: a review of the empirical evidence. Transp. Res. Part F Traffic Psychol. Behav. 27, 196–217. 10.1016/j.trf.2014.06.016

[B51] Durantin G. Dehais F. Delorme A. (2015). Characterization of mind wandering using fNIRS. Front. Syst. Neurosci. 9:45. 10.3389/fnsys.2015.00045 25859190 PMC4374461

[B52] Durso F. T. Hackworth C. A. Truitt T. R. Crutchfield J. Nikolic D. (1999). Situation Awareness As a Predictor of Performance in En Route Air Traffic Controllers. DTIC Document. Available online at: http://oai.dtic.mil/oai/oai?verb=getRecord&metadataPrefix=html&identifier=ADA360807 (Accessed August 23, 2016).

[B53] Dussault C. Jouanin J.-C. Philippe M. Guezennec C.-Y. (2005). EEG and ECG changes during simulator operation reflect mental workload and vigilance. Aviat. Space Environ. Med. 76, 344–351. 15828633

[B54] Eastwood J. D. Frischen A. Fenske M. J. Smilek D. (2012). The unengaged mind: defining boredom in terms of attention. Perspect. Psychol. Sci. 7, 482–495. 10.1177/1745691612456044 26168505

[B55] Eggemeier T. F. Wilson G. F. (1991). Performance-based and subjective assessment of workload in multi-task environments, in Multiple-Task Performance, ed Damos D. L. (London: Taylor & Francis), 217–278.

[B56] Endsley M. R. (1988). Situation awareness global assessment technique (SAGAT), in Aerospace and Electronics Conference, (1988). NAECON 1988., Proceedings of the IEEE 1988 National (IEEE), 789–795. Available at: http://ieeexplore.ieee.org/xpls/abs_all.jsp?arnumber=195097 (Accessed May 25, 2016).

[B57] Endsley M. R. Kiris E. O. (1995). The out-of-the-loop performance problem and level of control in automation. Hum. Factors 37, 381–394. 10.1518/001872095779064555

[B58] Endsley M. R. Rodgers M. D. (1998). Distribution of attention, situation awareness & workload in a passive air traffic control task. Air Traffic Control Q. 6, 21–44. 10.2514/atcq.6.1.21

[B59] Esterman M. Noonan S. K. Rosenberg M. DeGutis J. (2013). In the Zone or Zoning Out? Tracking behavioral and neural fluctuations during sustained attention. Cereb. Cortex 23, 2712–2723. 10.1093/cercor/bhs261 22941724

[B60] Federal Aviation Authority (1972). Eastern Airlines Flight 401, L-1011, Accident near Miami - Accident Overview. Lessons Learn. Available online at: http://lessonslearned.faa.gov/ll_main.cfm?TabID=1&LLID=8 (Accessed September 20, 2016).

[B61] Federal Aviation Authority (1995). American Airlines Flight 965, B-757, Accident near Cali - Accident Overview. Available online at: http://lessonslearned.faa.gov/ll_main.cfm?TabID=3&LLID=43(Accessed August 18, 2016).

[B62] Feng S. D'Mello S. Graesser A. C. (2013). Mind wandering while reading easy and difficult texts. Psychon. Bull. Rev. 20, 586–592. 10.3758/s13423-012-0367-y 23288660

[B63] Foxe J. J. Snyder A. C. (2011). The role of alpha-band brain oscillations as a sensory suppression mechanism during selective attention. Front. Psychol. 2:154. 10.3389/fpsyg.2011.00154 21779269 PMC3132683

[B64] Foxe J. J. Simpson G. V. Ahlfors S. P. (1998). Parieto-occipital ~10 Hz activity reflects anticipatory state of visual attention mechanisms. Neuroreport 9, 3929–3933. 10.1097/00001756-199812010-00030 9875731

[B65] Franklin M. S. Smallwood J. Schooler J. W. (2011). Catching the mind in flight: using behavioral indices to detect mindless reading in real time. Psychon. Bull. Rev. 18, 992–997. 10.3758/s13423-011-0109-6 21547521

[B66] Freeman F. G. Mikulka P. J. Scerbo M. W. Scott L. (2004). An evaluation of an adaptive automation system using a cognitive vigilance task. Biol. Psychol. 67, 283–297. 10.1016/j.biopsycho.2004.01.002 15294387

[B67] Galera C. Orriols L. M'Bailara K. Laborey M. Contrand B. Ribereau-Gayon R.. (2012). Mind wandering and driving: responsibility case-control study. BMJ 345, e8105–e8105. 10.1136/bmj.e8105 23241270 PMC3521876

[B68] Gerbert K. Kemmler R. (1986). The causes of causes: determinants and background variables of human factor incidents and accidents. Ergonomics 29, 1439–1453. 10.1080/00140138608967257 3803371

[B69] Gilbert S. J. Dumontheil I. Simons J. S. Frith C. D. Burgess P. W. (2007). Wandering minds: the default network and stimulus-independent thought. Science 317, 393–395. 10.1126/science.1140801 17615325

[B70] Gilzenrat M. S. Nieuwenhuis S. Jepma M. Cohen J. D. (2010). Pupil diameter tracks changes in control state predicted by the adaptive gain theory of locus coeruleus function. Cogn. Affect. Behav. Neurosci. 10, 252–269. 10.3758/CABN.10.2.252 20498349 PMC3403821

[B71] Golchert J. Smallwood J. Jefferies E. Seli P. Huntenburg J. M. Liem F.. (2016). Individual variation in intentionality in the mind-wandering state is reflected in the integration of the default-mode, fronto-parietal, and limbic networks. Neuroimage 146, 226–235. 10.1016/j.neuroimage.2016.11.025 27864082

[B72] Grandchamp R. Braboszcz C. Delorme A. (2014). Oculometric variations during mind wandering. Front. Psychol. 5:31. 10.3389/fpsyg.2014.00031 24575056 PMC3920102

[B73] Grandy T. H. Werkle-Bergner M. Chicherio C. Schmiedek F. Lövdén M. Lindenberger U. (2013). Peak individual alpha frequency qualifies as a stable neurophysiological trait marker in healthy younger and older adults: alpha stability. Psychophysiology 50, 570–582. 10.1111/psyp.12043 23551082

[B74] Hancock P. A. Williams G. (1993). Effect of task load and task load increment on performance and workload, in Seventh International Symposium on Aviation Psychology (Columbus, OH).

[B75] Harris M. B. (2000). Correlates and characteristics of boredom proneness and boredom. J. Appl. Soc. Psychol. 30, 576–598. 10.1111/j.1559-1816.2000.tb02497.x

[B76] Harris W. C. Hancock P. A. Arthur E. (1993). The effect of taskload projection on automation use, performance and workload, in Seventh International Symposium on Aviation Psychology (Columbus, OH).

[B77] He J. Becic E. Lee Y.-C. McCarley J. S. (2011). Mind wandering behind the wheel: performance and oculomotor correlates. Hum. Factors 53, 17–21. 10.1177/0018720810391530 21469530

[B78] Helton W. S. Warm J. S. (2008). Signal salience and the mindlessness theory of vigilance. Acta Psychol. 129, 18–25. 10.1016/j.actpsy.2008.04.002 18499079

[B79] Hu N. He S. Xu B. (2012). Different efficiencies of attentional orienting in different wandering minds. Conscious. Cogn. 21, 139–148. 10.1016/j.concog.2011.12.007 22248446

[B80] Jepma M. Nieuwenhuis S. (2011). Pupil diameter predicts changes in the exploration–exploitation trade-off: evidence for the adaptive gain theory. J. Cogn. Neurosci. 23, 1587–1596. 10.1162/jocn.2010.21548 20666595

[B81] Jeroski J. Miller M. E. Langhals B. Tripp L. (2014). Impact of vigilance decrement upon physiology measures, in IIE Annual Conference. Proceedings (Institute of Industrial Engineers-Publisher), 1184 Available online at: http://www.xcdsystem.com/iie2014/abstract/finalpapers/I471.pdf (Accessed December 2, 2015).

[B82] Kaber D. B. Onal E. Endsley M. R. (2000). Design of automation for telerobots and the effect on performance, operator situation awareness, and subjective workload. Hum. Factors Ergon. Manuf. Serv. Ind. 10, 409–430. 10.1002/1520-6564(200023)10:4<409::AID-HFM4>3.0.CO;2-V

[B83] Kam J. W. Y. Dao E. Blinn P. Krigolson O. E. Boyd L. A. Handy T. C. (2012). Mind wandering and motor control: off-task thinking disrupts the online adjustment of behavior. Front. Hum. Neurosci. 6:329. 10.3389/fnhum.2012.00329 23248596 PMC3522104

[B84] Kam J. W. Y. Dao E. Farley J. Fitzpatrick K. Smallwood J. Schooler J. W.. (2011). Slow fluctuations in attentional control of sensory cortex. J. Cogn. Neurosci. 23, 460–470. 10.1162/jocn.2010.21443 20146593

[B85] Kane M. J. Brown L. H. McVay J. C. Silvia P. J. Myin-Germeys I. Kwapil T. R. (2007). For whom the mind wanders, and when an experience-sampling study of working memory and executive control in daily life. Psychol. Sci. 18, 614–621. 10.1111/j.1467-9280.2007.01948.x 17614870

[B86] Khan M. J. Hong K.-S. (2015). Passive BCI based on drowsiness detection: an fNIRS study. Biomed. Opt. Express 6:4063. 10.1364/BOE.6.004063 26504654 PMC4605063

[B87] Killingsworth M. A. Gilbert D. T. (2010). A wandering mind is an unhappy mind. Science 330, 932–932. 10.1126/science.1192439 21071660

[B88] Konishi M. McLaren D. G. Engen H. Smallwood J. (2015). Shaped by the past: the default mode network supports cognition that is independent of immediate perceptual input. PLoS ONE 10:e0132209. 10.1371/journal.pone.0132209 26125559 PMC4488375

[B89] Kramer A. F. (1991). Physiological metrics of mental workload: a review of recent progress, in Multiple-Task Performance, ed Damos D. L. (London: Taylor & Francis), 279–328.

[B90] Lee J. D. See K. A. (2004). Trust in automation: designing for appropriate reliance. Hum. Factors 46, 50–80. 10.1518/hfes.46.1.50.30392 15151155

[B91] Lerner N. Baldwin C. Higgins J. S. Lee J. Schooler J. (2015). Mind wandering while driving: what does it mean and what do we do about it? Proc. Hum. Factors Ergon. Soc. Annu. Meet. 59, 1686–1690. 10.1177/1541931215591364

[B92] Lorenz B. Di Nocera F. Roettger S. Parasuraman R. (2002). Automated fault management in a simulated space flight micro-world. Aviat. Space Environ. Med. 73, 886–897. 12234040

[B93] Lowenstein O. Loewenfeld I. E. (1962). The pupil, in The eye, ed Davson H. (New York, NY: Academic Press), 231–267.

[B94] Luck S. J. (2014). An Introduction to the Event-Related Potential Technique. Cambridge: MIT Press.

[B95] Mackworth N. H. (1948). The breakdown of vigilance during prolonged visual search. Q. J. Exp. Psychol. 1, 6–21. 10.1080/17470214808416738

[B96] Manly T. Robertson I. H. Galloway M. Hawkins K. (1999). The absent mind: further investigations of sustained attention to response. Neuropsychologia 37, 661–670. 10.1016/S0028-3932(98)00127-4 10390027

[B97] Manzey D. Bahner J. E. Hüper A.-D. (2006). Misuse of automated aids in process control: Complacency, automation bias and possible training interventions, in Proceedings of the Human Factors and Ergonomics Society Annual Meeting (Sage, CA; Los Angeles, CA: Sage Publications), 220–224. Available online at: http://journals.sagepub.com/doi/abs/10.1177/154193120605000303 (Accessed March 6, 2017).

[B98] Mason M. F. Norton M. I. Van Horn J. D. Wegner D. M. Grafton S. T. Macrae C. N. (2007). Wandering minds: the default network and stimulus-independent thought. Science 315, 393–395. 10.1126/science.1131295 17234951 PMC1821121

[B99] Mathewson K. E. (2011). Pulsed Out of Awareness: EEG Alpha Oscillations Represent a Pulsed Inhibition of Ongoing Cortical Processing. Available online at: https://www.ideals.illinois.edu/handle/2142/26295 (Accessed May 23, 2016). 10.3389/fpsyg.2011.00099 PMC3132674 21779257

[B100] Matthews M. D. Beal S. A. (2002). Assessing Situation Awareness in Field Training Exercises. DTIC Document. Available online at: http://oai.dtic.mil/oai/oai?verb=getRecord&metadataPrefix=html&identifier=ADA408560 (Accessed August 23, 2016).

[B101] May J. F. Baldwin C. L. (2009). Driver fatigue: the importance of identifying causal factors of fatigue when considering detection and countermeasure technologies. Transp. Res. Part F Traffic Psychol. Behav. 12, 218–224. 10.1016/j.trf.2008.11.005

[B102] McMillan R. L. Kaufman S. B. Singer J. L. (2013). Ode to positive constructive daydreaming. Front. Psychol. 4:626. 10.3389/fpsyg.2013.00626 24065936 PMC3779797

[B103] McRoy S. (2017). Detecting, Repairing and Preventing Human-Machine Miscommunication. Available online at: http://tigger.cs.uwm.edu/~mcroy/mnm-si/ (Accessed August 2, 2017).

[B104] McVay J. C. Kane M. J. (2009). Conducting the train of thought: working memory capacity, goal neglect, and mind wandering in an executive-control task. J. Exp. Psychol. Learn. Mem. Cogn. 35, 196. 10.1037/a0014104 19210090 PMC2750806

[B105] McVay J. C. Kane M. J. (2010). Does mind wandering reflect executive function or executive failure? Comment on Smallwood and Schooler (2006) and Watkins (2008). Psychol. Bull. 136, 188–207. 10.1037/a0018298 20192557 PMC2850105

[B106] Melinscak F. Montesano L. Minguez J. (2014). Discriminating between attention and mind wandering during movement using EEG, in Proceedings of the 6th International Brain-Computer Interface Conference. Available online at: https://www.researchgate.net/profile/Filip_Melinscak/publication/268518939_Discriminating_Between_Attention_and_Mind_Wandering_During_Movement_Using_EEG/links/546f24660cf24af340bf848e.pdf (Accessed May 3, 2016).

[B107] Merat N. Jamson A. H. (2008). The effect of stimulus modality on signal detection: implications for assessing the safety of in-vehicle technology. Hum. Factors 50, 145–158. 10.1518/001872008X250656 18354978

[B108] Methot L. L. Huitema B. E. (1998). Effects of signal probability on individual differences in vigilance. Hum. Factors 40, 102–110. 10.1518/001872098779480514 9579106

[B109] Metzger U. Parasuraman R. (2001). The role of the air traffic controller in future air traffic management: an empirical study of active control versus passive monitoring. Hum. Factors 43, 519–528. 10.1518/001872001775870421 12002002

[B110] Mikulka P. J. Scerbo M. W. Freeman F. G. (2002). Effects of a biocybernetic system on vigilance performance. Hum. Factors 44, 654–664. 10.1518/0018720024496944 12691372

[B111] Mittner M. Boekel W. Tucker A. M. Turner B. M. Heathcote A. Forstmann B. U. (2014). When the brain takes a break: a model-based analysis of mind wandering. J. Neurosci. 34, 16286–16295. 10.1523/JNEUROSCI.2062-14.2014 25471568 PMC4252543

[B112] Mkrtchyan A. A. Macbeth J. C. Solovey E. T. Ryan J. C. Cummings M. L. (2012). Using variable-rate alerting to counter boredom in human supervisory control. Proc. Hum. Factors Ergon. Soc. Annu. Meet. 56, 1441–1445. 10.1177/1071181312561406

[B113] Molloy R. Parasuraman R. (1996). Monitoring an automated system for a single failure: vigilance and task complexity effects. Hum. Factors 38, 311–322. 10.1177/001872089606380211 11536753

[B114] Mooneyham B. W. Schooler J. W. (2013). The costs and benefits of mind-wandering: a review. Can. J. Exp. Psychol. Can. Psychol. Exp. 67, 11–18. 10.1037/a0031569 23458547

[B115] Moray N. (1986). Monitoring behavior and supervisory control, in Handbook of Perception and Human Performance, Vol. 2; Cognitive Processes and Performance, ed Boff K. (New York, NY: Wiley), 1–51.

[B116] Moray N. Inagaki T. (2000). Attention and complacency. Theor. Issues Ergon. Sci. 1, 354–365. 10.1080/14639220052399159

[B117] Morrison J. G. Cohen D. Gluckman J. P. (1993). Prospective principles and guidelines for the design of adaptively automated crewstations, in Seventh International Symposium on Aviation Psychology (Columbus, OH).

[B118] Mosier K. L. Skitka L. J. Korte K. J. (1994). Cognitive and social psychological issues in flight crew/automation interaction, in Human Performance in Automated Systems: Current Research and Trends, eds Mouloua M. Parasuraman R. (New York, NY: Cambridge University Press), 191–197.

[B119] Mullen T. R. Kothe C. A. E. Chi Y. M. Ojeda A. Kerth T. Makeig S.. (2015). Real-time neuroimaging and cognitive monitoring using wearable dry EEG. IEEE Trans. Biomed. Eng. 62, 2553–2567. 10.1109/TBME.2015.2481482 26415149 PMC4710679

[B120] National Transport Safety Board (1975). Aircraft Accident Report, Northwest Airlines Incorporated, Boeing 727-25, N26 4US near Thiells, New York, December 1st 1974. National Transport Safety Board.

[B121] Naujoks F. Purucker C. Neukum A. (2016). Secondary task engagement and vehicle automation – comparing the effects of different automation levels in an on-road experiment. Transp. Res. Part F Traffic Psychol. Behav. 38, 67–82. 10.1016/j.trf.2016.01.011

[B122] O'Connell R. G. Dockree P. M. Robertson I. H. Bellgrove M. A. Foxe J. J. Kelly S. P. (2009). Uncovering the neural signature of lapsing attention: electrophysiological signals predict errors up to 20 s before they occur. J. Neurosci. 29, 8604–8611. 10.1523/JNEUROSCI.5967-08.2009 19571151 PMC6665670

[B123] O'Hanlon J. F. (1981). Boredom: practical consequences and a theory.pdf. Acta Psychol. 49, 52–82. 7304249 10.1016/0001-6918(81)90033-0

[B124] OpenBCI (2016). OpenBCI- Open Source Biosensing Tools (EEG, EMG, EKG, and More). Available online at: http://openbci.com/ (Accessed May 23, 2016).

[B125] Overgaard M. Fazekas P. (2016). Can no-report paradigms extract true consciousness.pdf. Trends Cogn. Sci. 20, 241–242. 10.1016/j.tics.2016.01.004 26880396

[B126] Parasuraman R. (1979). Memory load and event rate control sensitivity decrements in sustained attention. Science 205, 924–927. 10.1126/science.472714 472714

[B127] Parasuraman R. (1987). Human-computer monitoring. Hum. Factors 29, 695–706. 10.1177/001872088702900609

[B128] Parasuraman R. Riley V. (1997). Humans and automation: use, misuse, disuse and abuse. Hum. Factors 39, 230–253. 10.1518/001872097778543886

[B129] Parasuraman R. Wickens C. D. (2008). Humans: still vital after all these years of automation. Hum. Factors 50, 511–520. 10.1518/001872008X312198 18689061

[B130] Parasuraman R. Molloy R. Singh I. L. (1993a). Performance consequences of automation-induced “complacency.” Int. J. Aviat. Psychol. 3, 1–23. 10.1207/s15327108ijap0301_1

[B131] Parasuraman R. Mouloua M. Molloy R. (1996). Effects of adaptive task allocation on monitoring of automated systems. Hum. Factors 38, 665–679. 10.1518/001872096778827279 11536753

[B132] Parasuraman R. Mouloua M. Molloy R. Hilburn B. (1993b). Adaptive function allocation reduces performance costs of static automation, in Seventh International Symposium on Aviation Psychology (Columbus, OH).

[B133] Pham P. Wang J. (2015). AttentiveLearner: improving mobile mOOC learning via implicit heart rate tracking, in Artificial Intelligence in Education, eds Conati C. Heffernan N. Mitrovic A. Verdejo M. F. (Cham: Springer International Publishing), 367–376. Available online at: http://link.springer.com/10.1007/978-3-319-19773-9_37 (Accessed March 1, 2016).

[B134] Prinzel L. J. Parasuraman R. Freeman F. G. Scerbo M. W. Mikulka P. J. Pope A. T. (2003). Three Experiments Examining the Use of Electroencephalogram, Event-Related Potentials, and Heart-Rate Variability for Real-Time Human-Centered Adaptive Automation Design. National Aeronautics and Space Administration.

[B135] Qu W. Ge Y. Xiong Y. Carciofo R. Zhao W. Zhang K. (2015). The relationship between mind wandering and dangerous driving behavior among Chinese drivers. Saf. Sci. 78, 41–48. 10.1016/j.ssci.2015.04.016

[B136] Reichle E. D. Reineberg A. E. Schooler J. W. (2010). Eye movements during mindless reading. Psychol. Sci. 21, 1300–1310. 10.1177/0956797610378686 20679524

[B137] Salmon P. M. Stanton N. A. Walker G. H. Jenkins D. Ladva D. Rafferty L. (2009). Measuring situation awareness in complex systems: comparison of measures study. Int. J. Ind. Ergon. 39, 490–500. 10.1016/j.ergon.2008.10.010

[B138] Sarter N. B. (2000). The need for multisensory interfaces in support of effective attention allocation in highly dynamic event-driven domains: the case of cockpit automation. Int. J. Aviat. Psychol. 10, 231–245. 10.1207/S15327108IJAP1003_02

[B139] Sarter N. B. Woods D. D. (1995a). “From tool to agent”: the evolution of (Cockpit) automation and its impact on human-machine coordination, in Proceedings of the Human Factors and Ergonomics Society Annual Meeting (SAGE Publications), 79–83. Available online at: http://pro.sagepub.com/content/39/1/79.short (Accessed August 19, 2016).

[B140] Sarter N. B. Woods D. D. (1995b). How in the world did we ever get into that mode? Mode error and awareness in supervisory control. Hum. Factors 37, 5–19. 10.1518/001872095779049516

[B141] Sarter N. B. Woods D. D. Billings C. E. (1997). Automation surprises. Handb. Hum. Factors Ergon. 2, 1926–1943. 10.1518/001872097778667997 11536850

[B142] Scanella S. Peysakhovich V. Ehrig F. Dehais F. (2015). Can flight phase be inferred using eye movements? Evidence from real flight conditions, in 18th European Conference on Eye Movements (Vienna).

[B143] Schad D. J. Nuthmann A. Engbert R. (2012). Your mind wanders weakly, your mind wanders deeply: objective measures reveal mindless reading at different levels. Cognition 125, 179–194. 10.1016/j.cognition.2012.07.004 22857818

[B144] Schooler J. W. Mrazek M. D. Franklin M. S. Baird B. Mooneyham B. W. Zedelius C. (2014). The middle way, in Psychology of Learning and Motivation (Elsevier), 1–33. Available online at: http://linkinghub.elsevier.com/retrieve/pii/B9780128000908000019 (Accessed May 3, 2016).

[B145] Schooler J. W. Smallwood J. Christoff K. Handy T. C. Reichle E. D. Sayette M. A. (2011). Meta-awareness, perceptual decoupling and the wandering mind. Trends Cogn. Sci. 5, 319–326. 10.1016/j.tics.2011.05.006 21684189

[B146] Seli P. Risko E. F. Smilek D. (2016). On the necessity of distinguishing between unintentional and intentional mind wandering. Psychol. Sci. 1:7 10.1177/0956797616634068 26993740

[B147] Sheridan T. B. (1992). Telerobotics, Automation, and Human Supervisory Control. Cambridge: MIT Press.

[B148] Smallwood J. (2010). Why the global availability of mind wandering necessitates resource competition: reply to McVay and Kane (2010). Psychol. Bull. 136, 202–207. 10.1037/a0018673

[B149] Smallwood J. (2011). Mind-wandering while reading: attentional decoupling, mindless reading and the cascade model of inattention. Lang. Linguist. Compass 5, 63–77. 10.1111/j.1749-818X.2010.00263.x

[B150] Smallwood J. Schooler J. W. (2006). The restless mind. Psychol. Bull. 132, 946–958. 10.1037/0033-2909.132.6.946 17073528

[B151] Smallwood J. Schooler J. W. (2015). The science of mind wandering: empirically navigating the stream of consciousness. Annu. Rev. Psychol. 66, 487–518. 10.1146/annurev-psych-010814-015331 25293689

[B152] Smallwood J. Baracaia S. F. Lowe M. Obonsawin M. (2003). Task unrelated thought whilst encoding information. Conscious. Cogn. 12, 452–484. 10.1016/S1053-8100(03)00018-7 12941287

[B153] Smallwood J. Brown K. S. Tipper C. Giesbrecht B. Franklin M. S. Mrazek M. D.. (2011). Pupillometric evidence for the decoupling of attention from perceptual input during offline thought. PLoS ONE 6:e18298. 10.1371/journal.pone.0018298 21464969 PMC3064669

[B154] Smallwood J. Davies J. B. Heim D. Finnigan F. Sudberry M. O'Connor R.. (2004). Subjective experience and the attentional lapse: Task engagement and disengagement during sustained attention. Conscious. Cogn. 13, 657–690. 10.1016/j.concog.2004.06.003 15522626

[B155] Smallwood J. Fishman D. J. Schooler J. W. (2007). Counting the cost of an absent mind: mind wandering as an underrecognized influence on educational performance. Psychon. Bull. Rev. 14, 230–236. 10.3758/BF03194057 17694906

[B156] Smallwood J. McSpadden M. Schooler J. W. (2008). When attention matters: the curious incident of the wandering mind. Mem. Cogn. 36, 1144–1150. 10.3758/MC.36.6.1144 18927032

[B157] Smallwood J. Riby L. Heim D. Davies J. B. (2006). Encoding during the attentional lapse: accuracy of encoding during the semantic sustained attention to response task. Conscious. Cogn. 15, 218–231. 10.1016/j.concog.2005.03.003 16115782

[B158] Smilek D. Carriere J. S. A. Cheyne J. A. (2010). Failures of sustained attention in life, lab, and brain: ecological validity of the SART. Neuropsychologia 48, 2564–2570. 10.1016/j.neuropsychologia.2010.05.002 20452366

[B159] Smith R. P. (1981). Boredom: a review. Hum. Factors 23, 329–340. 10.1177/001872088102300308 7016721

[B160] Stawarczyk D. Majerus S. Van der Linden M. D'Argembeau A. (2012). Using the daydreaming frequency scale to investigate the relationships between mind-wandering, psychological well-being, and present-moment awareness. Front. Psychol. 3:363. 10.3389/fpsyg.2012.00363 23055995 PMC3457083

[B161] Strauch B. (2002). Investigating Human error: Incidents, Accidents, and Complex Systems. Burlington, VT: Ashgate.

[B162] Taheri B. A. Knight R. T. Smith R. L. (1994). A dry electrode for EEG recording. Electroencephalogr. Clin. Neurophysiol. 90, 376–383. 10.1016/0013-4694(94)90053-1 7514984

[B163] Taylor R. M. (1990). Situational Awareness Rating Technique (SART): The Development of a Tool for Aircrew Systems Design. AGARD situational aware. Aerosp. Oper. 17 PSEE N 90-28972 23–53.

[B164] Teasdale J. D. Dritschel B. H. Taylor M. J. Proctor L. Lloyd C. A. Nimmo-Smith I.. (1995). Stimulus-independent thought depends on central executive resources. Mem. Cogn. 23, 551–559. 10.3758/BF03197257 7476241

[B165] Teichner W. H. (1974). The detection of a simple visual signal as a function of time of watch. Hum. Factors 16, 339–352. 10.1177/001872087401600402 4435787

[B166] Thackray R. I. Touchstone R. M. (1989). A Comparison of Detection Efficiency on an Air Traffic Control Monitoring Task with and without Computer Aiding. US Departement of Transportation - Federal Aviation Administration Available online at: http://oai.dtic.mil/oai/oai?verb=getRecord&metadataPrefix=html&identifier=ADA206422 (Accessed February 26, 2016). 2775130

[B167] This Place (2016). MindRDR. Available online at: http://mindrdr.thisplace.com/static/index.html (Accessed May 23, 2016).

[B168] Thomson D. R. Seli P. Besner D. Smilek D. (2014). On the link between mind wandering and task performance over time. Conscious. Cogn. 27, 14–26. 10.1016/j.concog.2014.04.001 24780348

[B169] Tsuchiya N. Frässle S. Wilke M. Lamme V. (2016). No-report and report-based paradigms jointly unravel the NCC: response to Overgaard and Fazekas. Trends Cogn. Sci. 20, 241–242. 10.1016/j.tics.2016.01.006 26899261

[B170] Uzzaman S. Joordens S. (2011). The eyes know what you are thinking: eye movements as an objective measure of mind wandering. Conscious. Cogn. 20, 1882–1886. 10.1016/j.concog.2011.09.010 21968150

[B171] van Charante E. M. Cook R. I. Woods D. D. Yue L. Howie M. B. (1993). Human-computer interaction in context: physician interaction with automated intravenous controllers on the heart, in Analysis, Design and Evaluation of Man–Machine Systems 1992 (Elsevier), 263–274. Available online at: http://linkinghub.elsevier.com/retrieve/pii/B978008041900850044X (Accessed August 19, 2016).

[B172] van den Heuvel M. P. Hulshoff Pol H. E. (2010). Exploring the brain network: a review on resting-state fMRI functional connectivity. Eur. Neuropsychopharmacol. 20, 519–534. 10.1016/j.euroneuro.2010.03.008 20471808

[B173] van Vugt M. K. Taatgen N. A. Sackur J. Bastian M. Borst J. Mehlhorn K. (2015). Modeling mind-wandering: a tool to better understand distraction, in Proceedings of the 13th International Conference on Cognitive Modeling, eds van Vugt M. K. Borst J. Mehlhorn K. (Groningen: University of Groningen), 252.

[B174] Warm J. S. (1984). Sustained Attention in Human Performance. Chichester: Wiley.

[B175] Warm J. S. Dember W. N. Hancock P. A. (1996). Vigilance and workload in automated systems, in Automation and Human Performance: Theory and Applications, eds Parasuraman R. Mouloua M. (Mahwah, NJ: Lawrence Erlbaum), 183–200.

[B176] Warm J. S. Parasuraman R. Matthews G. (2008). Vigilance requires hard mental work and is stressful. Hum. Factors 50, 433–441. 10.1518/001872008X312152 18689050

[B177] Wickens C. D. Kessel C. (1977). The Effects of Participatory Mode and Task Workload on the Detection of Dynamic System Failures. Champaign, IL: Aviation Research Laboratory.

[B178] Wickens C. Dixon S. Goh J. Hammer B. (2005). Pilot Dependence on Imperfect Diagnostic Automation in Simulated UAV Flights: An Attentional Visual Scanning Analysis. DTIC Document. Available online at: http://oai.dtic.mil/oai/oai?verb=getRecord&metadataPrefix=html&identifier=ADA446167 (Accessed March 6, 2017).

[B179] Wiener E. L. (1987). Application of vigilance research: rare, medium, or well done? Hum. Factors 29, 725–736. 10.1177/001872088702900611 3325400

[B180] Wiener E. L. (1988). Cockpit Automation. National Aeronautics and Space Administration. Available online at: http://ntrs.nasa.gov/search.jsp?R=19890047073.

[B181] Willems B. Truitt T. R. (1999). Implications of Reduced Involvement In en Route Air Traffic Control. DTIC Document. Available at: http://oai.dtic.mil/oai/oai?verb=getRecord&metadataPrefix=html&identifier=ADA369584 (Accessed August 19, 2016).

[B182] Wise J. A. Tilden D. S. Abbott D. Dyck J. Guide P. (1994). Managing automation in the cockpit, in International Federation of Airworthiness, 24th International Conference. Available online at: http://www.faa.gov/training_testing/training/media/cfit/volume2/pdf/pages/page5_07.pdf (Accessed October 29, 2015).

[B183] Woods D. D. Tinapple D. (1999). W3: Watching human factors watch people at work, in 43rd Annual Meeting of the Human Factors Ergonomics and Society (Houston, TX).

[B184] Yanko M. R. Spalek T. M. (2014). Driving with the wandering mind: the effect that mind-wandering has on driving performance. Hum. Factors 56, 260–269. 10.1177/0018720813495280 24689247

[B185] Yoss R. E. Moyer N. J. Hollenhorst R. W. (1970). Pupil size and spontaneous pupillary waves associated with alertness, drowsiness, and sleep. Neurology 20, 545–554. 10.1212/WNL.20.6.545 5463609

[B186] Zedelius C. M. Schooler J. W. (2015). Mind wandering “Ahas” versus mindful reasoning: alternative routes to creative solutions. Front. Psychol. 6:834. 10.3389/fpsyg.2015.00834 26136715 PMC4469818

[B187] Zedelius C. M. Schooler J. W. (2016). The richness of inner experience: relating styles of daydreaming to creative processes. Front. Psychol. 6:2063. 10.3389/fpsyg.2015.02063 26869943 PMC4735674

